# A gender-specific assessment of tobacco use risk factors: evidence from the latest Pakistan demographic and health survey

**DOI:** 10.1186/s12889-022-13574-2

**Published:** 2022-06-06

**Authors:** Faiqa Zubair, Muhammad Iftikhar ul Husnain, Ting Zhao, Hasnat Ahmad, Rasheda Khanam

**Affiliations:** 1 Department of Economics, COMSATS University, Islamabad, 45550 Pakistan; 2 Menzies Institute for Medical Research, University of Tasmania, Hobart, 7000 Australia; 3 School of Business, University of Southern Queensland, Toowoomba, QLD 4350 Australia

**Keywords:** Logistic regression, Risk factors, Media exposure, Domestic violence, Tobacco smoking

## Abstract

**Background:**

The high prevalence of tobacco use in Pakistan poses a substantial health and economic burden to Pakistani individuals, families, and society. However, a comprehensive assessment of the key risk factors of tobacco use in Pakistan is very limited in the literature. A better understanding of the key risk factors of tobacco use is needed to identify and implement effective tobacco control measures.

**Objectives:**

To investigate the key socioeconomic, demographic, and psychosocial determinants of tobacco smoking in a recent large nationally representative sample of Pakistani adults.

**Methods:**

*N* = 18,737 participants (15,057 females and 3680 males) from the 2017–18 Pakistan Demographic Health Survey, aged 15–49 years, with data on smoking use and related factors were included. Characteristics of male and female participants were compared using T-tests (for continuous variables) and χ2-tests (for categorical variables). Multivariable logistic regression models were used to identify gender-specific risk factors of tobacco use. The Receiver Operating Characteristic Curve test was used to evaluate the predictive power of models.

**Results:**

We found that the probability of smoking for both males and females is significantly associated with factors such as their age, province/region of usual residence, education level, wealth, and marital status. For instance, the odds of smoking increased with age (from 1.00 [for ages 15–19 years] to 3.01 and 5.78 respectively for females and males aged 45–49 years) and decreased with increasing education (from 1.00 [for no education] to 0.47 and 0.50 for females and males with higher education) and wealth (from 1.00 [poorest] to 0.43 and 0.47 for richest females and males). Whilst the odd ratio of smoking for rural males (0.67) was significantly lower than that of urban males (1.00), the odds did not differ significantly between rural and urban females. Finally, factors such as occupation type, media influence, and domestic violence were associated with the probability of smoking for Pakistani females only.

**Conclusions:**

This study identified gender-specific factors contributing to the risk of tobacco usage in Pakistani adults, suggesting that policy interventions to curb tobacco consumption in Pakistan should be tailored to specific population sub-groups based on their sociodemographic and psychosocial features.

**Supplementary Information:**

The online version contains supplementary material available at 10.1186/s12889-022-13574-2.

## Background

Tobacco smoking is a major public health issue and results in the death of over 7 million active smokers and over 1 million passive smokers worldwide annually [1–5]. It is therefore one of the world’s principal causes of preventable deaths. Tobacco is an addictive drug that is abused in developing countries amongst the rural and urban populations [6]. Tobacco use significantly impacts individuals’ physical and psychosocial health-related quality of life (HRQoL) as well as posing a significant economic burden to smokers, their families and society in terms of associated direct (healthcare costs of treating tobacco use-related illnesses) and indirect costs (from lost productivity due to tobacco-attributable poor HRQoL) [7, 8]. Between 1960 and 2020, the diseases caused by tobacco smoking led to 41 million cumulative deaths in Canada, the United Kingdom and the United States [9]. In Pakistan, tobacco related diseases result in the death of > 100,000 people every year [10]*.*

Pakistani individuals are among the world’s largest consumers of tobacco and Pakistan ranks among the top 15 countries in the world in terms of the disease burden associated with tobacco smoking [11, 12] According to recent estimates, among adults aged 15 years and above, 27.0% malesand 5.5%females were recorded as daily tobacco users. In developing nations such as Pakistan, a multitude of factors (e.g., low tobacco prices, lack of awareness about negative effects of tobacco use, growth in population and aggressive tobacco marketing) determines continuous growth in tobacco usage, especially in youth and women. High prevalence of tobacco use in Pakistan poses a substantial health and economic burden to Pakistani individuals, their families and society. In fact, it could be considered to be a tobacco epidemic. National and international observers are concerned that, contrary to its commitment to reduce the prevalence of tobacco usage by 30% by 2025, the consumption of tobacco in Pakistan may increase [3]. The increasing prevalence of the tobacco epidemic has made it an urgent and clear public health priority around the globe based on its substantial economic and health consequences [13].

The extant literature has identified a number of factors related to tobacco consumption. These include sociodemographic factors such as age, gender, marital status, place of usual residence, occupation, social and religious affiliations and economic status [14, 15], school-related factors such as influence of peers, smoking friends, low academic performance, school disapproval, truancy level, weak student-teacher relationships and students’ perception of tobacco availability at school [16, 17], and other factors such as the use of illicit drugs, early sexual activity, and low levels of physical activity [1, 18]. The limited numbers of studies [12, 19–21] that have investigated the risk factors for tobacco use in the Pakistani population have suffered from several limitations. First, mass media plays an important role in influencing the audience to quit smoking or encouraging the audience to begin smoking. However, no previous study in Pakistan has analysed the effects of anti-smoking media campaigns on smoking cessationusing national level data. Second, no study, to date, has investigated the relationship between domestic violence (recognized as a cultural norm in a male dominated Pakistani society) and smoking behaviour. Finally, other studies that investigated the determinants of smoking in Pakistan were either based on data that is now more than a decade old and/or their analysis was restricted to data collected from Pakistani males only [12, 19, 22]. This study aims to address these research gaps by using the most recent nationally representative data set from Pakistan.

In the light of above-mentioned discussion, the prime objective of the study is to assess gender specific tobacco use risk factors in Pakistan with a special focus on anti-smoking media campaigns and domestic violence by using most recent available data from Pakistan Demographic and Health Survey. The importance of this study is manyfold: first, use of tobacco is considerably increasing in Pakistan that may lead to serious health consequences in future which warrants narrow investigation of the determinants of smoking. Second, domestic violence is prevalent especially in rural areas which may inflate tobacco use which suggest assessment of the nexus between domestic violence and smoking behaviour in a patriarchal society. Finally, it is of paramount importance to see the impact of anti-smoking campaigns on the smoking behaviour in Pakistan.

## Materials and methods

### Study population

The data used in this study came from nationally representative Pakistan Demographic and Health Survey (PDHS) 2017–18 which provided information on many health indicators including socioeconomic determinants and information on tobacco use. Data collection for the PDHS 2017–18 occurred between 22 November 2017 and 30 April 2018. The PDHS 2017–18 is Pakistan’s fourth Demographic and Health Survey, following the PDHS 1990–91, PDHS 2006–07, and PDHS 2012–13. The PDHS 2017–18 collected the required data from both the rural and urban areas of Pakistan’s four provinces (i.e., Punjab, Sindh, Khyber Pakhtunkhwa, and Baluchistan) as well as the regions outside of the four provinces (Azad Jammu and Kashmir [AJK], Gilgit Baltistan [GB], Islamabad Capital Territory [ICT] and Federally Administered Tribal Areas [FATA]). Importantly, this is the first time in the history of PDHS that populations of AJK and FATA are covered. The sampling frame used for the 2017–18 PDHS is a comprehensive list of enumeration blocks (EBs). A stratified two-stage sample model was adopted by the PDHS 2017–18. A maximum of 16 sampling strata were formed after separating each one of the eight regions (Punjab, Sindh, Khyber Pakhtunkhwa, Baluchistan, ICT, GB, AJK and FATA) into urban and rural domains. A sum of 580 clusters (sampling points) was identified at the first level. A household listing procedure was conducted in all chosen clusters and 28 households per cluster were chosen for a maximum sample size of 16,240 households.

The 2017–18 PDHS comprised six questionnaires: 1) Household Questionnaire, 2) Woman’s Questionnaire, 3) Man’s Questionnaire, 4) Biomarker Questionnaire, 5) Fieldworker Questionnaire, and 6) Community Questionnaire. The first five questionnaires were tailored to represent the population and health concerns applicable to Pakistan, relying on the standard Demographic and Health Survey (DHS-7) questionnaires of the DHS Program. Fundamental sociodemographic data (including age, sex, education, marital status, and relation to household head) were collected from all survey participants. Ever married men (*n* = 3145 [one-third of the selected households]) and women (*n* = 12, 364) aged 15–49 years were asked questions about having knowledge, attitudes, and behaviour related smoking and other health issues (e.g., tuberculosis, hepatitis.

### Measurement of variables

Many factors can influence tobacco use that make choice of variables a challenging task. The choice of variables in this study is based on the literature review and the context of Pakistan. For example, age, area, region, education level and marital status are extensively used in tobacco related empirical literature. Whereas domestic violence and media exposure are two highly important variables in the context of Pakistan. In recent past media has become an independent entity and strong pillar of state. However, anti-smoking campaigns were not sufficiently effective in reducing tobacco use in Pakistan. Likewise, domestic violence is frequently prevalent in Pakistani society that may determine tobacco behaviour in the country.

In the regression analysis, smoking status is the dependent variable. Participants were asked if they are currently smoking tobacco, with the answer choices of ‘yes’ and ‘no’. Those answering ‘yes’ to this question were categorised as smokers and others as non-smokers. Whereas age, province/region of usual residence, education level, wealth, occupation, marital status, media exposure and domestic violence are the independent variables. Dependent variable indicates the failure or success of smoking behaviour. The probability of smoking is “1” (*p* = P(smoke = 1)) and whereas, probability of not smoking is “0” (*p* = 1-P(no smoke = 0)).

Detailed characteristics of smokers by gender are summarized in Table 1.

**Table 1:** Detailed characteristics of smokers by gender

Age group (Years)	Males		Females	
	smokers (n)	smokers (%)	smokers (n)	smokers (%)
15–24 years	94	7.5%	191	14.8%
25–29 years	202	16.0%	250	19.5%
30–34 years	238	18.8%	230	17.9%
35–39 years	271	21.4%	241	18.8%
40–44 years	220	17.4%	180	14.0%
45–49 years	239	18.9%	191	14.9%
Total	1264	100.0%	1283	100.0%
**Province/ Region**				
Punjab	303	24.0%	185	14.4%
Sindh	260	20.6%	436	34.0%
KPK	165	13.1%	68	5.3%
Baluchistan	111	8.8%	415	32.3%
GB	66	5.2%	51	4.0%
ICT	109	8.6%	47	3.7%
AJK	148	11.7%	67	5.2%
FATA	102	8.1%	14	1.1%
Total	1264	100.0%	1283	100.0%
**Area**				
Urban	668	52.8%	532	41.5%
Rural	596	47.2%	751	58.5%
Total	1264	100.0%	1283	100.0%
**Educational Level**				
No education	337	26.7%	920	71.7%
Primary	248	19.6%	141	11.0%
Secondary	485	38.4%	152	11.9%
Higher	194	15.4%	70	5.5%
Total	1264	100%	1283	100.0%
**Economic Status**				
Poorest	255	20.2%	425	33.1%
Poorer	283	22.4%	327	25.5%
Middle	268	21.2%	246	19.2%
Richer	258	20.4%	171	13.3%
Richest	200	15.8%	114	8.9%
Total	1264	100.0%	1283	100.0%
**Occupation group**				
Not working	32	2.5%	1008	78.6%
Officers/ Managers	151	11.9%	24	1.9%
Clerical staff	1081	85.5%	251	19.6%
Total	1264	100.0%	1283	100.0%
**Marital Status**				
Married	1240	98.1%	1213	94.5%
Others*	24	1.9%	70	5.4%
Total	1264	100.0%	1283	100.0%
**Media Access**				
Not at all	766	60.6%	577	45.0%
Less than once a week	498	39.4%	676	52.7%
At least once a week	0	0.0%	30	2.3%
Total	1264	100.0%	1283	100.0%
**Domestic Violence**				
No	766	60.6%	577	45.0%
Yes	498	39.4%	676	52.7%
Don’t know	0	0.0%	30	2.3%
Total	1264	100.0%	1283	100.0%

Notes: KPK (Khyber Pakhtunkhwa), GB (Gilgit Baltistan), ICT (Islamabad Capital Territory), AJK (Azad Jammu and Kashmir), FATA (Federally Administered Tribal Areas. *Others category of marital status included participants that are divorced, widowed and those no longer living together

Among the total sample of smokers, 1264 are male and 1283 are female. The highest percentage of male smokers is in the age group of 35–39 years (21.4%), belongs to the Punjab province (24%), lives in urban areas (52.8%), have 12 years of education (secondary [38.4%]), belongs to poor families, has a clerical job (85.5%), is married (98.1%), has no access to media (60.6%), and never experienced domestic violence (60.6%). On the other hand, the highest percentage of women smokers are relatively young (25–29 [19.5%]), belong to Sindh province (34%), live in a rural area (58.5%), are illiterate (71.7%), have the poorest economic status (33.1%), are not working (78.6%), are married (94.5%), have limited access to media (less than once a week [52.7%]), and have experienced domestic violence (52.7%).

Univariate analyses were conducted using Pearson Chi-square Tests (χ2-test)/Fisher’s exact tests to compare the differences between categorical variables. The multi-variable logistic regressions were used to identify the association between smoking behaviour and various sociodemographic characteristics in both sexes. Furthermore, the study employed likelihood ratio which is a worth considering tool to assess evidence in data about two competing a priori hypotheses. The procedure of this test is analogous to the general linear F test procedure which tests overall significance of the model in multiple linear regression. In evidence evaluation, the likelihood ratio test quantifies the magnitude of the evidence in favor of null or the alternate proposition by considering the conditional probability of each proposition and then Bayesian prior odds are converted into the posterior odds.

In health-related analysis, statistical tests and *p*-values are subject to high criticism mainly because users misunderstand these methods. However, there are very few alternatives to these statistical techniques. Confidence intervals estimate the parameters of interest and are used increasingly however, considered substitutes of tests. An appealing alternative is Bayesian analysis which has limited use as it radically departures from status quo. In contrast likelihood ratio is a worth considering tool to assess determinants of a health issues which enable researcher to quantify the support of one hypothesis over the other. The Hosmer-Lemeshow test (HL test) was used to test the goodness of fit for the logistic regression model to check how well our data fits the model. Additionally, a Receiver Operating Characteristic (ROC) Curve was used to evaluate diagnostics and the predictive power of models.

## Results

### Univariate analyses

The background characteristics of the study population by smokers and non-smokers are reported in Table 2 which shows that the percentage of both the men and women smokers is increasing with age. For instance, the percentage of smokers in both genders (12% for men and 5% for women) is at its lowest in the 15–19 years age bracket and highest (40% for men and 12% for women) in those aged 45–49 years. Table 2 also reveals that FATA is recorded for having the highest percentage of male smokers at 46%, followed by 44% in AJK, 41% in ICT, 36% in Punjab, 33% in Sindh and KPK, 31% in GB and 21% in Baluchistan. Interestingly, the proportion of female smokers at 1% was the lowest in FATA and highest in Baluchistan (24%), followed by Sindh (16%), Punjab (5%), GB (5%), AJK and ICT (4%), and KPK (3%).

**Table 2:** Percent distribution of men and women, smokers, and non-smokers – Pearson Chi square Test

Characteristics	Men		n	Women		n
	non-smokers	smokers		non-smokers	smokers	
**Age group**	Χ^2^ = 36.10			Χ^2^ = 51.74		
15˗19 years	87.76	12.24	49	94.64	5.36	728
20˗24 years	69.97	30.03	293	93.15	6.85	2219
25˗29 years	69.81	30.19	669	92.05	7.95	3143
30–34 years	68.77	31.23	762	91.93	8.07	2851
35–39 years	63.03	36.97	733	91.19	8.81	2736
40–44 years	62.07	37.93	580	90.11	9.89	1820
45–49 years	59.76	40.24	594	87.76	12.24	1560
**Province/Region**	Χ^2^ = 73.20			Χ^2^ = 1.0e + 02		
Punjab	64.35	35.65	850	94.55	5.45	3396
Sindh	66.54	33.46	777	84.08	15.92	2738
KPK	67.26	32.74	504	97.14	2.86	2377
Baluchistan	78.53	21.47	517	75.90	24.10	1722
GB	68.57	31.43	210	94.82	5.18	984
ICT	58.71	41.29	264	95.76	4.24	1108
AJK	55.95	44.05	336	96.10	3.89	1720
FATA	54.05	45.95	222	98.62	1.38	1012
**Area**	Χ^2^ = 2.38			Χ^2^ = 24.95		
Urban	64.47	35.53	1880	92.66	7.34	7247
Rural	66.89	33.11	1800	90.38	9.62	7810
**Educational Level**	Χ^2^ = 74.10			Χ^2^ = 266.37		
No education	61.09	38.91	866	87.93	12.07	7624
Primary	60.38	39.62	626	93.29	6.71	2101
Secondary	63.29	36.71	1321	95.14	4.86	3130
Higher	77.62	22.38	867	96.82	3.18	2202
**Economic Status**	Χ^2^ = 36.03			Χ^2^ = 270.46		
Poorest	61.94	38.06	670	85.27	14.73	2885
Poorer	64.49	35.51	797	89.91	10.09	3240
Middle	62.09	37.91	707	91.70	8.30	2963
Richer	64.32	35.68	723	94.05	5.95	2875
Richest	74.46	25.54	783	96.32	3.68	3094
**Occupation group**	Χ^2^ = 27.30			Χ^2^ = 83.69		
Not working	72.88	27.12	118	92.09	7.91	12,745
Officers/ Managers	74.28	25.72	587	95.31	4.69	512
Clerical staff	63.66	36.34	2975	86.06	13.94	1800
**Marital Status**	Χ^2^ = 7.26			Χ^2^ = 15.22		
Married	65.72	34.28	3617	91.63	8.37	14,492
Widowed	60.87	39.13	23	88.77	11.23	365
Divorced	76.92	23.08	26	87.50	12.50	136
No longer living together	35.71	64.29	14	81.25	18.75	64
**Media Access**	Χ^2^ = 3.77			Χ^2^ = 37.07		
Not at all	63.33	36.67	990	90.01	9.99	6109
Less than once a week	65.29	34.71	628	90.63	9.38	1632
At least once a week	66.88	33.12	2062	92.89	7.11	7316
**Domestic Violence**	Χ^2^ = 12.82			Χ^2^ = 60.41		
No	67.58	32.42	2363	92.99	7.00	8236
Yes	62.10	37.90	1314	89.8	10.20	6628
Don’t know	100	0.00	3	84.46	15.54	193

Notes: KPK (Khyber Pakhtunkhwa), GB (Gilgit Baltistan), ICT (Islamabad Capital Territory), AJK (Azad Jammu and Kashmir), FATA (Federally Administered Tribal Areas)

Rural Pakistani women were found to be more likely to smoke (9.62%) than urban women (7.34%). However, the proportion of male smokers did not significantly differ between rural and urban areas. The proportion of male smokers was the highest in those living in urban areas (36%), with a primary education (40%), earning poor income (38%), working as clerks (36%), separated from their spouses (64%), and having no exposure to any kind of media (37%). However, the difference between rural males and urban males was found to be statistically insignificant. In contrast, the highest proportion of female smokers was living in rural areas (10%), uneducated (12%), having the lowest income level (15%), working as clerks (14%), separated (19%) and with no kind of media access (10%). Men who committed domestic violence also tended to be more likely to smoke tobacco (38%) as compared to those who did not commit domestic violence (32%). On the other hand, women exposed to domestic violence were more likely to smoke (10%) than those not exposed to domestic violence (7%).

### Results from the multivariate logistic regression models

Table 3 shows that the odds of smoking for both men and women were significantly associated with age, province/region of usual residence, education level, wealth, marital status, and media exposure. For instance, the odds of smoking significantly increased with age (from 1.00 for men and women aged 15–19 years to 5.78 (for men) and 3.01 (for women) aged 45–49 years. Moreover, the odds of smoking for men were higher than those for women at all age groups (Table 3).

**Table 3:** Odds ratio of men and women smoking

Variables	LR Test for Men	LR Test for Women
	***P*** -value < 0.001	***P***-value < 0.001
**Age group**		
15–19 years	0.00 [Reference]	0.00 [Reference]
20–24 years	3.49*** (1.41,8.61)	1.55** (1.06, 2.26)
25–29 years	3.66*** (1.51,8.87)	1.89*** (1.32, 2.72)
30–34 years	3.71*** (1.53,8.97)	2.05*** (1.42, 2.95)
35–39 years	4.78*** (1.98,11.55)	2.08*** (1.44, 2.99)
40–44 years	5.21*** (2.14,12.64)	2.53*** (1.74, 3.69)
45–49 years	5.77*** (2.38,13.99)	3.00*** (2.06, 4.37)
**Province/Region of usual residence**		
Punjab	0.00 [Reference]	0.00 [Reference]
Sindh	0.88 (0.70,1.10)	3.22*** (2.65, 3.90)
KPK	0.86 (0.67,1.10)	0.51*** (0.38, 0.69)
Baluchistan	0.42*** (0.32, 0.55)	4.59*** (3.74, 5.64)
GB	0.84 (0.59,1.18)	0.80 (0.57, 1.12)
ICT	1.60*** (1.18,2.16)	1.11* (0.79, 1.55)
AJK	1.39** (1.06,1.82)	0.75* (0.56, 1.00)
FATA	1.51** (1.08,2.11)	0.18*** (0.10, 0.31)
**Area of residence**		
Urban	0.00 [Reference]	0.00 [Reference]
Rural	0.66*** (0.56,0.78)	1.09 (0.94, 1.26)
**Educational Level No education 0.00 [Reference]**		**0.00 [Reference]**
Primary	0.92 (0.73,1.15)	0.80** (0.65, 0.98)
Secondary	0.83* (0.67,1.01)	0.67*** (0.54, 0.83)
Higher	0.50*** (0.38,0.65)	0.46*** (0.34, 0.63)
**Economic Status**		
Poorest	0.00 [Reference]	0.00 [Reference]
Poorer	0.84 (0.67,1.07)	0.86* (0.72, 1.02)
Middle	0.84 (0.65,1.09)	0.81* (0.66, 1.00)
Richer	0.75** (0.56,0.99)	0.58*** (0.45, 0.74)
Richest	0.47*** (0.34,0.65)	0.43*** (0.32, 0.58)
**Occupation**		
**Unemployed**	0.00 [Reference]	0.00 [Reference]
Officers/ Managers/Professionals	1.10 (0.68,1.77)	1.34 (0.84, 2.15)
Clerks/Workers	1.25 (0.81,1.95)	1.18** (1.00, 1.40)
**Marital Status**		
**Married**	0.00 [Reference]	0.00 [Reference]
Widowed	0.90 (0.37,2.18)	1.15 (0.81, 1.64)
Divorced	0.55 (0.21,1.40)	1.72* (0.99, 2.97)
Separated	2.88* (0.91,9.10)	3.19*** (1.63, 6.24)
**Media Access**		
**Not at all**	0.00 [Reference]	0.00 [Reference]
Less than once a week	1.12 (0.89,1.42)	1.04 (0.85, 1.28)
At least once a week	1.09 (0.89,1.32)	1.18** (1.01, 1.38)
**Domestic Violence**		
**No**	0.00 [Reference]	0.00 [Reference]
Yes	1.10 (0.94,1.30)	1.26*** (1.10, 1.43)
Don’t know		1.36 (0.88, 2.09)
**Constant**	0.191***	0.034***

Notes: (1) ‘*’, **., *** indicates 10, 5% & 1% level of significance respectively; (2) () Confidence interval at 95% level of significance

It was found that males living in Islamabad were more likely to smoke (odd ratio1.6) than males living in all other regions whilst women living in Baluchistan, the least developed province, were more likely to smoke (odd ratio 4.56) than women living elsewhere in the country. Conversely, men living in Baluchistan were least likely to smoke than men living elsewhere in the country. Unexpectedly, the odds of smoking for women living in rural area (1.09) were higher than men living in rural areas (0.66). Economic status significantly determined smoking behaviour in males as well as in females and the probability of being a smoker decreased as the economic status of respondents improved from poorer (0.85) to richer (from 0.85 to 0.47 for men and from 0.86 to 0.43 for women). The odds of smoking for men were higher than women at all economic status excluding the status of “poorer”. Access to media was not significant for smoking behaviour in men whilst for females, it showed that women with more media access were more likely to smoke (odd ratio 1.18) when compared with women who were less exposed to media. Domestic violence was not statistically significant for the smoking behaviour of men while women facing domestic violence were more likely to smoke (odds ratio 1.26) as compared to women who did not experience domestic violence. Moreover, the odds of smoking for women exposed to domestic violence (1.26) were higher than those of men having domestic violence (1.10).

Educational status of both Pakistani men and women was strongly linked with smoking. Pakistani men and women with secondary or higher level of education were less likely to smoke than those having no education (secondary education, odds ratio for men and women are 0.83 and 0.67, respectively; higher education odds ratio for men and women are 0.50 and 0.46, respectively). Moreover, the odds of smoking for men were higher than those for women at all levels of education.

Furthermore, separated men and women were more likely to smoke, having odds ratio of 2.88 and 3.19 respectively compared to those men and women who were married. Also, the odds of smoking for separated women (3.19) were higher than for separated men (2.88). Furthermore, the odds of smoking for women were higher at all levels of marital status when compared with men.

The Hosmer Lemeshow Test values for men and women at 8 degrees of freedom are 8.15 (*p* = 0.4186) and 14.63 (*p* = 0.06) respectively, showing that the model is specified correctly. Supplementary Fig. 1 shows the Receiver Operating Curve Test (ROC) that graphically represents the prediction power of the estimated models for men (Part a) and women (Part b). Supplementary Fig. 1 Parts (a) and (b) both represent the fair prediction power of the estimated models in the study as the area under the ROC curve is 0. 65 and 0.77 for men and women, respectively.

To test whether each of the determinant of smoking behaviour significantly contributes to the model, we perform likelihood ratio test. Due to wide acceptance of likelihood ratio tests, they are considered as an automatic solution to testing problems. As the significance of the test is small i.e., less than 0.05, it is concluded that each factor is significantly associated with the smoking behaviour.

## Discussion

The present study investigates socio-economic and psychosocial factors and media exposure as the determinants of tobacco consumption among Pakistani men and women. Findings are based on the data collected from the Pakistan Demographic and Health Survey of 2017–18 which is sufficiently reliable and representative to provide an ample opportunity to the investigate tobacco smoking behavior of Pakistani adults. For the first time, this study focused on the association between media exposure and smoking using national level data in Pakistan and investigated the relationship between domestic violence (recognized as a cultural norm in a male dominated Pakistani society) and smoking behavior. The study reveals that socioeconomic (age, region, wealth, education level and marital status) and psychosocial conditions (domestic violence) are the key determinants of of tobacco smoking for both males and females in Pakistan.

Contrary to previous empirical literature, Pakistani men and women tended to increase smoking as they age. Previous studies on smoking in Pakistan are scarce. However, the finding that older people smoke more is in accordance with work from other developing and developed countries [1, 23, 24]. Furthermore, people in other countries in Asia having similar stages of economic development to those of Pakistan are exposed to similar risks and the findings of this study can be equally applicable to such countries. Smoking prevalence among aged people is likely to increase in Pakistan in the near future, revealing the high risk of a smoking epidemic. Results have demonstrated the negative association between education level and tobacco consumption which is in line with previous literature on the subject [14, 22, 25–29].

Smoking patterns differ with area of residence, not only in Pakistan but in comparable countries. It was revealed that FATA has the highest percentage of men smokers (46%) which can be attributed to the high incidence of poverty and low literacy rate in these areas. In contrast, the percentage of women smokers in FATA is the lowest because of cultural norms that restrict women’s activities outside home and thus afford them a lack of exposure to harmful behaviour like smoking. Men who smoke tend to live in urban areas (36%). Rural Pakistani women are found to be more involved in smoking (9.62%) in comparison to urban women (7.34%). Poverty is endemic in rural areas of Pakistan and is a causative factor in the prevalence of smoking among women due to their poor economic condition. This finding is supported by dose-response relationship which states that smoking is significantly associated with lower levels of income worldwide [30]. In comparable countries such as Latin America and the Caribbean, an inverse relationship was observed between tobacco prevalence and income level [31]. A positive relationship between tobacco use and residential rural area is found by Alam, Iqbal [32] in the case of Pakistan. Nasir, Imran [20] reported the region as a significant determinant of tobacco smoking. Rahman, Mondal [33] reported a high smoking prevalence of male population living in rural areas of Bangladesh. In Indonesia the proportion of smokers living in rural areas was higher than in urban areas [34].

The odds values showing the probabilities of smoking for men and women who are appointed as officers/ managers are insignificant. The probability of women who are working as clerks (odds ratio 1.18) is that they are more likely to smoke than those who are not working. Both men and women working in non-professional labour sectors have a higher probability of smoking because they have less awareness of the risks associated with tobacco use and a work environment that makes the use of tobacco more acceptable. By observing that education determines smoking behaviour, this non-professional labour might be particularly exposed to tobacco usage risk. In addition to their having less awareness of the health risks, their work environment that makes tobacco consumption more acceptable may encourage them to initiate smoking [35]. However, in literature these trends are inconsistent and related to costs associated with different types of tobacco use. The individuals working as officers/ managers may consume tobacco less often but when they do, they tend to consume expensive types of tobacco.

The results showed that exposure to media has its impact on the smoking habits of Pakistani men and women, supporting the empirical findings that different media exposures are associated with a higher likelihood of the escalation of tobacco smoking [36, 37]. For the Indian population, Paul and Majumdar [4] found a higher prevalence of tobacco smoking where people have access to radio and television. However, this finding is not compatible with the view that mass media exposure does not influence tobacco usage in Afghanistan [29]. The finding that domestic violence against women increases tobacco consumption is in line with the findings of previous literature [23, 38–40], that reports a similar conclusion in the case of India and other low to middle income countries. Intimate partner violence results in many mental and physical problems in women to which they may respond by resorting to more smoking.

Furthermore, separated men and women are more likely to smoke than those men and women who are married and living together. The odds of smoking for separated women (3.19) is more than for separated men (2.88) as separated women alone carry much of the socio-economic family cost and face hardships in the patriarchal society of Pakistan; therefore, to find relief from such burdens they resort to unhealthy activities like tobacco smoking. These results are in line with those of Caleyachetty, Echouffo-Tcheugui [39] who found a positive relationship between domestic violence and prevalence of tobacco smoking in pregnant women. In a similar study, Santaularia, Johnson [41] demonstrated that individuals having experienced sexual violence at early stages of their life are more inclined to smoke later in life.

This study complements and extends previous empirical findings by demonstrating gender-specific assessment of tobacco consumption risk factors using large nationally representative dataset. Our work highlights the importance of gender-specific intervention efforts and anti-smoking campaigns to curb increasing trend of smoking in Pakistan. While tailoring policies, it is of paramount importance to consider complex interplay of several risk factors associated with smoking including, age, level of education, area of residence, marital status, exposure to domestic violence, economic status, occupation, and access to media. A strong governmental commitment and backing will be essential in ensuring the complete success of various anti-smoking policies, programs, and campaigns.

Several limitations suggest that the findings of this study should be interpreted with caution. First, the cross-sectional design of the study constrains deducing any type of causal inference between smoking and its determinants. Second, many unmeasured and unobserved factors (e.g., frequency of media exposure, the cost of tobacco products, the individual’s perception about tobacco usage and knowledge about its harmful health effects) may have an influence on the findings. Stigma associated with tobacco smoking especially in women may have led to under-reporting the rate of tobacco use. Third, the survey does not put forward contextual information about an individual’s tobacco smoking behaviour. Fourth, no concrete information is available as to how social media and internet influence smoking. Fifth, this study focusses on only tobacco smoking while other modes of smoking are also typical among the population. Sixth, the possibility of type 1 error may question these findings, given the fact that variables included in the model may be highly correlated. Finally, due to the significant time lag between data collection and documentation of these results, there might be change in current realities. Despite these constraints the conclusions of this paper are sufficiently compelling to provide insights for useful policy frameworks about the use of tobacco use in Pakistan.

## Conclusion

This study has shown that socioeconomic factors such as age, education, region, type of place of residence, marital status, occupation, media access, economic status, and domestic violence are strong predictors of smoking behaviour in both males and females in Pakistan. To help minimize smoking practices in females, intervention may need to be targeted at women living in rural areas and having faced domestic violence. Likewise, males residing in urban areas and with no access to media must be targeted. Moreover, men as well as women with low-income levels should be targeted on priority basis. This may be achieved, by running hard-hitting awareness campaigns about the harmful effects of smoking that may provide a protective factor against smoking behaviour. In light of the findings of this study, it is also suggested that potential efforts are mandatory where the literacy rate is poor and where inadequate media reporting is prominent, especially in remote areas, so the adverse effects of cigarette smoking to one’s health could be minimized. To protect women from domestic violence, household members should also be helped, warned, and treated with awareness programs. Strategies must be framed to strengthen coordination between prevention and support services, in the context of humanized approach to women’s health, for women who experience domestic violence and consume tobacco. The pro-poor policies need to be framed to increase income level of the poor through different economic programmes like provision of micro credit to start small businesses. Furthermore, the findings of this study are believed to support health professionals in controlling and managing cases of violence by knowing their associated factors. The prime objective of this study was to assess the gender-specific tobacco use risk factors in Pakistan with a special focus on anti-smoking media campaigns and domestic violence. The careful attention toward findings reached in this study reveals that the said objective was comprehensively achieved.

## Supplementary Information

**Additional file 1: Supplementary figure 1.** Receiver Operating Curve Test for (a) men and (b) women

## Data Availability

The data that support the findings of this study are available from the Demographic and Health Surveys (DHS) Program at https://dhsprogram.com/methodology/survey/survey-display-523.cfm. Restrictions apply to the availability of these data, as available only to the registered/approved users. The data that support the findings of this study are also available from the corresponding author (IH) with permission from the DHS, upon reasonable request.
