# Supplementary material for: A gender-specific assessment of tobacco use risk factors: evidence from the latest Pakistan demographic and health survey
Source: BMC Public Health. 2022 Jun 6;22:1133. doi: 10.1186/s12889-022-13574-2 (PMC9172179; doi:10.1186/s12889-022-13574-2)
Supplement: Supplementary file 1 — Additional file 1: Supplementary figure 1. Receiver Operating Curve Test for (a) men and (b) women [file 12889_2022_13574_MOESM1_ESM.docx]

**Supplementary figure 1: Receiver Operating Curve Test for (a) men and (b) women**

(a)(b)
